# Research on Synthetic Data Methods and Detection Models for Micro-Cracks

**DOI:** 10.3390/s26061883

**Published:** 2026-03-17

**Authors:** Yaotong Jiang, Tianmiao Wang, Xuanhe Chen, Jianhong Liang

**Affiliations:** 1 School of Mechanical Engineering and Automation, Beihang University, Beijing 100191, China; yaotong1995@buaa.edu.cn (Y.J.); itm@buaa.edu.cn (T.W.); 2 School of Physical Science and Technology, Beijing University of Posts and Telecommunications, Beijing 102206, China; 2010815252@bupt.cn

**Keywords:** micro-crack detection, concrete inspection, Poisson image editing, synthetic data, sparse transformer, small-object enhancement, real-time detection

## Abstract

**Highlights:**

**What are the main findings?**

- A Poisson image editing-based synthesis pipeline produces visually consistent micro-crack samples and improves data availability without degrading realism.
- The proposed CST-YOLO (YOLOv10 + LAPM + SCS-Former + SOFB) achieves 0.926 mAP@0.5:0.95 at 139 FPS on real-image evaluation, with complementary gains verified by ablations.

**What are the implications of the main findings?**

- The combined “data realism + architecture robustness” strategy enables accurate and efficient micro-crack detection under challenging illumination, shadow, and clutter conditions.
- The real-time performance supports practical deployment in large-scale infrastructure inspection scenarios that require both precision and throughput.

**Abstract:**

Micro-crack detection on concrete surfaces is challenging because labeled micro-crack data are scarce, crack cues are extremely weak (often only a few pixels wide), and complex backgrounds (e.g., non-uniform illumination, shadows, and stains) degrade feature extraction; this study aims to improve both data availability and detection robustness for practical inspection. A Poisson image editing-based synthesis strategy is developed to generate visually coherent micro-crack samples via gradient-domain blending, and a Complex-Scene-Tolerant YOLO (CST-YOLO) detector is proposed on top of YOLOv10, following an “lighting decoupling–global perception–micro-feature enhancement” design. CST-YOLO integrates an Lighting-Adaptive Preprocessing Module (LAPM) to suppress illumination/shadow perturbations, a Spatial–Channel Sparse Transformer (SCS-Former) to model long-range crack topology efficiently, and a Small Object Focus Block (SOFB) to enhance micro-scale cues under cluttered backgrounds. Experiments are conducted on a 650-image dataset (200 real and 450 synthesized), in which synthesized samples are used only for training, and the validation/test sets contain only real images, with a 7:2:1 split. CST-YOLO achieves 0.990 mAP@0.5 and 0.926 mAP@0.5:0.95 at 139 FPS, and ablation results indicate complementary contributions from LAPM, SCS-Former, and SOFB. These results support the effectiveness of combining realistic synthesis and architecture-level robustness for real-time micro-crack detection in complex scenes.

## 1. Introduction

Bridges, as key nodes in transportation networks, directly affect traffic safety and regional economic operations. As the service life of bridge structures increases, concrete materials inevitably develop micro-cracks, through cracks, and local damage such as surface defects due to long-term vehicle loads, temperature and humidity cycles, chloride salt corrosion, freeze–thaw cycles, and carbonation. Cracks not only serve as significant visible indicators of durability degradation but also as major pathways for water and harmful ions to penetrate, causing rebar corrosion and secondary damage. From an engineering maintenance perspective, information on the spatial distribution, orientation, width, and length of cracks can provide direct guidance for structural condition assessment, damage classification, repair and reinforcement decisions, and predictions of long-term evolution. Therefore, bridge concrete crack detection technologies for engineering applications must meet comprehensive requirements for high-precision identification, robustness in complex scenarios, the ability to quantify scale, and deployability to play a practical role in the “inspection-assessment-decision-closure” chain.

Traditional bridge inspections still heavily rely on manual close-range visual inspection, knocking, and measuring, which, while intuitive, have obvious limitations: (1) detection efficiency is limited, making it difficult to support high-frequency inspections of large-scale bridges; (2) results are influenced by the inspector’s experience, leading to subjectivity and poor repeatability; (3) work in high-risk areas such as the underside of beams poses significant risks and is constrained by traffic conditions; (4) data often lacks structured representation, making it difficult to form time-based traceable comparisons. To improve inspection efficiency and objectivity, researchers have proposed crack extraction methods based on image processing and feature engineering (e.g., threshold segmentation, edge detection, texture analysis), but in real-world bridge environments, cracks often present geometric and imaging characteristics such as “thin, weak contrast, and intermittent distribution”. Additionally, background textures, construction joints, leakage stains, repair marks, shadows, and reflections are common interferences that lead traditional methods to exhibit high false positives and missed detections in complex backgrounds, and they are sensitive to parameters, with limited cross-scenario generalization ability. In response, deep learning has become the mainstream approach for crack detection due to its end-to-end feature-learning capabilities [1,2,3].

From a broader application perspective, bridge crack detection shares commonalities with tasks such as road, tunnel, and building facade defect detection: all require identifying small defects in complex backgrounds while considering engineering efficiency. Therefore, in addition to bridge-specific research, studies on road cracks and multi-defect recognition have also provided transferable insights for bridge tasks. For example, multi-defect recognition research uses image fusion and deep networks to identify multiple types of surface defects, providing a “multi-task, multi-semantic” modeling perspective for bridge health assessment [4]; road crack detection research enhances the response to fine cracks and suppresses background texture interference from the perspectives of multi-stage feature fusion and texture perception [5]. Some studies have also introduced structural improvements to the YOLO framework to enhance road crack detection performance [6]. In tunnel and enclosed-space scenarios, low illumination and non-uniform lighting are more prevalent. Researchers have combined image enhancement (e.g., improved Retinex) with deep learning detection/segmentation to improve crack visibility and detection rates under weak light conditions [7]; other works have introduced no-reference image quality assessment (NR-IQA) to filter and analyze the quality of collected images, highlighting that degradation such as blur and compression significantly affects deep model detection performance [8]. These studies collectively indicate that data quality, scene variations, and scale changes are unavoidable engineering factors in surface defect and bridge crack detection, and that bridge crack detection requires system designs and method optimizations that are better aligned with real-world operational conditions.

At the data collection and operational platform level, bridge crack detection is trending towards “mobility, autonomy, and systematization”. Unmanned Aerial Vehicles (UAVs) have advantages such as wide coverage, flexible deployment, and strong accessibility to the underside of beams, making them suitable for rapid bridge inspection and large-scale modeling. Some studies have further coupled crack detection with 3D reconstruction to enable 3D visualization of crack distribution, supporting engineers in rapid localization and decision-making [9,10]. Compared with UAVs, ground-based mobile platforms or robotic inspections offer more stable close-range imaging conditions, providing advantages for fine re-measurements and repeated observations. Related research has proposed automated crack-monitoring systems that combine mobile robots with robotic arms and binocular vision, thereby improving the repeatability and automation of inspections [11]. However, differences in engineering platforms also introduce new challenges: UAVs face issues such as viewpoint variations, scale fluctuations, and motion-blur risks, whereas robotic/vehicle-mounted platforms are constrained by accessibility and occlusion. Therefore, crack detection algorithms must maintain robust performance across different platforms and imaging conditions while also balancing real-time feedback and edge-deployment capabilities.

Based on existing research, bridge concrete crack identification methods can generally be categorized into three task paradigms: (1) Classification and Multi-Defect Recognition, (2) Crack Object Detection, and (3) Pixel-Level Segmentation and Fine Measurement. To compare the applicability of different paradigms in engineering implementation, this paper summarizes the advantages and limitations of common technical approaches, as shown in Table 1.

The multi-defect recognition task focuses on the unified identification of various defects such as cracks, spalling, leakage, and stains, emphasizing the value of multi-semantic coverage for engineering inspections. Research combining image fusion with deep learning has provided an end-to-end solution for multi-defect surface recognition, thereby enhancing discrimination in complex backgrounds to some extent [4]. However, considering the core needs of bridge crack detection, merely performing category classification or coarse-grained recognition is insufficient to support crack localization and scale quantification. Additionally, the coexistence of multiple defects, inter-class similarities, and long-tail distributions often leads to false positives and missed detections. To address the issue of insufficient samples, data augmentation is widely adopted. Relevant studies have shown that augmentation strategies, such as GANs, can improve multi-class damage detection performance under certain conditions; however, discrepancies between augmented and real distributions may also introduce new generalization risks [12]. Therefore, multi-defect recognition is more suitable as a “rapid screening and alerting” module, while the engineering loop for bridge crack detection still requires further reliance on localization and fine measurement capabilities.

Object detection has an intuitive advantage in engineering inspections: it can quickly identify areas with cracks, providing candidate regions of interest (ROIs) for subsequent fine analysis, and is more suitable for real-time edge deployment. General object detection methods have evolved from two-stage detection (e.g., Faster R-CNN) to one-stage detection (e.g., the YOLO series). The former achieves strong localization via candidate regions and classification regression [20], while the latter, with end-to-end dense prediction, strikes a better balance between accuracy and speed, becoming a key branch for real-time detection [21]. Based on this, the YOLO series has continuously evolved: YOLOX expanded the performance boundaries of YOLO in training strategies and label assignment [22]; YOLOF explored a more lightweight feature layer design through the “one-level feature” idea [23]; YOLOv10 emphasized end-to-end and real-time performance [24]; YOLOv12 further discussed the efficient integration of attention mechanisms in the YOLO framework [25]. Meanwhile, the end-to-end DETR (Detection Transformers) system has also made progress in real-time detection. Research on RT-DETR shows that, through structural and training strategy design, DETR-based models can compete with YOLO-like methods in real-time scenarios [26].

In crack detection applications, a large body of work employs YOLO as the foundational framework, enhancing recall for small cracks and the ability to distinguish complex backgrounds through multi-scale feature fusion, attention mechanisms, and improved loss functions. In road crack detection, structural improvements based on YOLOv4 have been made, optimizing modules to address the “thin, small, and varied morphology” of cracks [6]. In real-time detection of sidewalk cracks with UAVs, researchers have conducted precision-speed trade-off analyses of different YOLO structures and discussed the impacts of shadows, rain, and blur [13]. Tunnel lining multi-defect detection studies emphasize rapid localization of multiple defects in complex texture and polluted backgrounds [27]. In bridge scenarios, the lightweight YOLOv4 algorithm enables edge deployment by reducing parameters and computational requirements [14], whereas YOLOv8-based models that incorporate attention mechanisms and improve the IoU loss further enhance the accuracy and generalization of bridge crack detection [15]. However, object detection naturally produces bounding-box-level results, making it difficult to accurately delineate the true boundaries and the connected structure of cracks. This poses an inherent constraint in engineering quantification, such as crack width/length, and implies that “improvements in detection metrics” do not necessarily correspond to “enhancements in crack geometric continuity”.

To meet engineering quantification needs, pixel-level segmentation has become an important direction in crack research. Segmentation methods can output continuous boundaries, providing a more direct data foundation for estimating crack geometry, connectivity, and width. Related reviews have systematically compared pavement crack segmentation methods, pointing out that segmentation has advantages in terms of precision but also places higher demands on annotation costs, network complexity, and low-light robustness [19]. In bridge scenarios, existing studies have used UAV imagery and deep learning for pixel-level crack identification, thereby laying the groundwork for crack boundary extraction and subsequent analysis [17]. Meanwhile, the detection–segmentation coupling strategy has become a practical compromise for engineering applications: it quickly locks in the region of interest (ROI) via detection and then performs fine segmentation in the local area. This approach reduces the cost of full-image segmentation while enhancing boundary delineation capabilities. A typical work proposed CR-YOLO for rapid crack localization, combined with an improved PSPNet for fine segmentation, and emphasized its potential for real-time detection and segmentation on edge devices [18]. Additionally, to achieve the “detection-quantification-visualization” loop, some studies have integrated multi-scale crack detection, crack width calculation, 3D modeling, and report generation, thereby enabling quantitative estimation of crack parameters and 3D mapping [10,28,29,30,31].

Engineering maintenance not only concerns “whether cracks exist,” but also focuses on “where the cracks are, how they are distributed, and whether they are expanding”. Therefore, mapping crack results to a 3D model or global coordinate system can significantly enhance the interpretability and traceability of the results. Existing studies have proposed crack detection and 3D visualization processes for UAV-based bridge inspections, thereby providing an intuitive representation of crack spatial distribution [9]. Other research has combined multi-scale crack detection with techniques such as SfM (Structure-from-Motion) and GCP (Ground Control Points) to construct verifiable 3D models for crack quantification and 3D mapping [10]. However, the 3D process is sensitive to pose estimation, reconstruction quality, and scale calibration, and errors may be propagated and magnified in the “detection-mapping-measurement” chain, making it more challenging to assess engineering reliability. Therefore, while 3D mapping is an important trend, it still requires integration with robust detection and uncertainty management to reliably support engineering decision-making [32,33,34].

Although the above research has pushed bridge concrete crack detection from “usable” to “more usable,” several common shortcomings still exist for real-world engineering deployment, constituting current research gaps:

1. Insufficient robustness under complex imaging degradation. Shadows, reflections, low illumination, motion blur, and compression noise are commonly present in bridge inspections and significantly reduce the contrast between cracks and the background. Studies such as NR-IQA have revealed that CNN model performance varies significantly across different quality thresholds [8]. However, most current work remains at the “enhancement/filtering” level, and there is no unified framework for collaboratively optimizing quality metrics, prediction uncertainty, and detection networks.
2. Continuity expression and multi-scale compatibility for small cracks remain bottlenecks. Cracks often occur in thin structures, and endpoint and bifurcation information can be easily lost. Scale variation under UAV perspectives is vast, making it difficult to balance “recall” and “false detection suppression” using a unified threshold and single-scale features. Multi-scale detection and quantification workflows can partially mitigate this issue [10], but when extremely thin cracks, complex textures, and non-uniform lighting overlap, cracks remain prone to being broken, missed, or misclassified as pseudo-cracks.
3. Cross-scenario generalization and data dependence are prominent issues. Significant differences exist in bridge surface weathering, pollutant types, and repair status, and there are notable domain differences relative to roads/tunnels and other scenarios. Models often rely on specific data distributions. Data augmentation can alleviate sample shortages and improve multi-class damage detection performance [12], but discrepancies between augmented distributions and real distributions may introduce biases. Furthermore, there are no guidelines for selecting augmentation strategies that account for crack “geometric priors,” leading to instability in cross-bridge type and cross-season generalization.
4. Lack of a systematic trade-off paradigm between real-time deployability and fine quantification. Object detection is well-suited to real-time deployment, but bounding-box results cannot support metrics such as width/length. Segmentation/3D mapping can be used for quantification and visualization, but the labeling, computational power, and engineering process complexity are high. Although lightweight designs (e.g., the lightweight YOLOv4 for bridges) have improved edge feasibility [14], there remains a lack of a unified, reproducible engineering evaluation and optimization framework under multi-constraint conditions.

Given the current state of research and identified gaps, this paper focuses on the bridge concrete surface crack detection task, aiming to enhance robustness in complex scenes and fine-crack recognition capabilities while ensuring engineering deployability. The main contributions of this work are summarized as follows:

1. Proposed a Poisson Image Editing-based micro-crack synthetic data pipeline to alleviate small sample and distribution inadequacy problems. To address the high annotation cost, scarcity of real samples, and difficulty in covering diverse backgrounds, this paper constructs a crack synthesis strategy based on gradient domain fusion (Poisson image editing), achieving natural transitions and texture consistency between the crack foreground and different concrete backgrounds. Compared to traditional copy-paste augmentation methods, this strategy improves the usability of training data without significantly reducing realism, thereby enhancing the model’s generalization ability at the data level.
2. Proposed CST-YOLO: An integrated framework for micro-crack detection in complex scenes, forming a design paradigm of “environment decoupling—global perception—micro-feature enhancement”. Building on the YOLOv10 detection framework, this paper introduces Complex-Scene-Tolerant YOLO (CST-YOLO), which addresses three key challenges: environmental disturbances, such as complex illumination/shadows; long-range dependencies in crack topology; and the loss of high-frequency details in small targets. These challenges are modeled and optimized hierarchically, thereby improving the separability and detection stability of microcracks in complex structural scenarios.
3. Designed and integrated three key modules (LAPM, SCS-Former, SOFB), corresponding to environmental robustness, long-range modeling, and micro-scale enhancement.
  - LAPM (Lighting-Adaptive Preprocessing Module): Inspired by Retinex theory, it suppresses illumination/shadow disturbances and stabilizes shallow feature distributions, achieving “environment decoupling”.
  - SCS-Former (Spatial-Channel Sparse Transformer): Efficiently captures the long-range continuity of crack elongation structures through spatial sparse modeling and channel recalibration, enhancing “global perception” while maintaining computational efficiency.
  - SOFB (Small Object Focus Block): Strengthens the expression of micro-crack details through high-frequency enhancement and robust channel attention, thereby alleviating background-dominated statistics arising from sparse small targets, thereby achieving “micro-feature enhancement”.
4. Achieved a balance between accuracy and speed in real image evaluation protocols, and verified effectiveness and complementary contributions through comparative and ablation experiments. Using a 650-image dataset with both real and synthetic samples, this paper employs an evaluation strategy where “synthetic data is used only for training, and validation/testing use real images,” and conducts systematic comparisons and ablation analyses. The results show that CST-YOLO achieves 0.990 mAP@0.5 and 0.926 mAP@0.5:0.95 on real-image evaluation, with real-time inference at 139 FPS. Ablation experiments validate the complementary gains of LAPM, SCS-Former, and SOFB, providing evidence for the feasibility of large-scale engineering inspection deployment.

## 2. Poisson Image Editing-Based Image Data Construction Method

In deep learning-based crack detection tasks for concrete structures, the scale, quality, and distribution characteristics of the training data largely determine the model’s generalization capability and stability. However, in real-world engineering scenarios, obtaining crack data is constrained by significant practical limitations. On the one hand, acquiring crack images with specific features and manually annotating them is costly, making it difficult to rapidly build large-scale datasets. On the other hand, the limited number of real samples is often concentrated in specific structural types or environmental conditions, resulting in a narrow data distribution that degrades performance when the model is applied to different scenes.

To alleviate the data bottleneck, researchers commonly adopt data augmentation techniques to expand the training sample space. However, most existing augmentation methods rely on geometric transformations or pixel-level stitching, which often exhibit significant deficiencies in visual continuity and physical plausibility. Specifically, they struggle to simulate the coupling relationship between cracks and the background lighting or material texture on real concrete surfaces. This “non-physically consistent” synthesis approach can introduce artificial artifacts, causing the model to learn edge or texture features unrelated to real cracks, thus weakening its generalization ability.

To address the above issues, this paper proposes a Poisson editing-based method for synthesizing crack images. Rather than simply copying and pasting crack samples, the method starts from the morphological features and imaging mechanisms of cracks. By combining morphological analysis with Poisson Image Editing theory [35], it naturally merges cracks under diverse lighting and grayscale distributions while preserving the authenticity of their internal texture and structure.

Let the source image (crack foreground) be S, and the target image (background) be T. The region where the crack is embedded into the target image is denoted as $\Omega \subset R^{2}$, with boundary 𝜕Ω, and the fusion result is I. The gradient domain fusion goal can be expressed as follows: within Ω, making ∇I as close as possible to ∇S, while satisfying the Dirichlet boundary condition on 𝜕Ω, ensuring consistency with the background.

The corresponding energy minimization model is

(1) $$\underset{I}{min}\;\int_{\Omega }\;{∥\nabla I(x)-\nabla S(x)∥}_{2}^{2}dx,\;s.t.I(x)=T(x),x\in 𝜕\Omega$$

where x=(x,y) is the spatial position variable, and ∇ is the gradient operator. The above equation represents constraints on any spatial position within the fusion region Ω (i.e., any pixel location) and applies boundary conditions to the pixel locations on the boundary 𝜕Ω. This modeling constrains the fusion result to approximate the crack foreground in terms of gradient while ensuring a seamless transition between the fusion region and the background at the boundary via the Dirichlet boundary condition.

In this modeling framework, the color value of the image is viewed as the value of a continuous spatial function at position  x and $\int_{\Omega }(\cdot )\;dx$ represents the overall accumulation over all spatial positions within the fusion region. In subsequent numerical implementations, this continuous integral form will be approximated by summation over a discrete pixel grid.

To validate the proposed fusion method and to facilitate the subsequent experimental verification of the innovations introduced in this study, we conducted on-site data collection and constructed an image dataset. Specifically, we captured nearly 800 high-resolution images containing real micro-cracks using the Sony A7M4 and the Fujifilm GFX100S camera, with resolutions of 33 megapixels and 100 megapixels, respectively. Because the acquired images were extremely large and thus unsuitable for direct use as a training dataset, we selected 500 images and randomly cropped crack foreground patches and background patches, followed by one-to-one fusion. Each background patch was fused with only a single crack foreground to avoid repeated synthesis on the same background. Importantly, the fusion procedure was performed under the constraint that the synthesized crack must not overlap with any existing cracks in the background.

**Figure 1:**
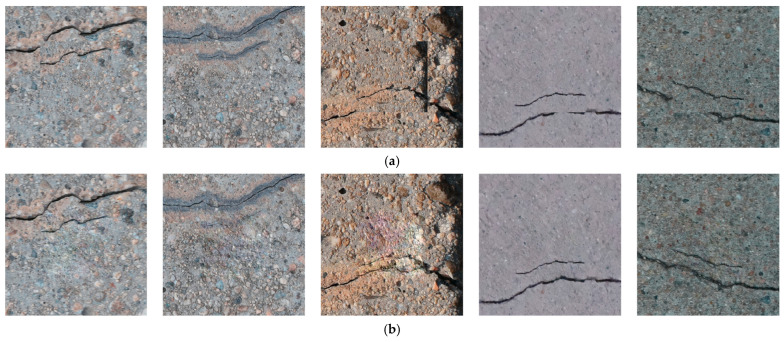
Comparison of Crack Image Fusion Results: Traditional Copy-Paste Method vs. Proposed Method: (**a**) Traditional Copy-Paste Method; (**b**) Proposed Method.

Figure 1 compares the traditional “copy-paste” method with the crack image generated using the Poisson editing-based data construction method proposed in this paper. The method presented in this paper clearly preserves the internal texture and structural authenticity of microcracks, in contrast to the traditional method. Additionally, at the background edges, it achieves a natural fusion in terms of lighting and grayscale distribution with the diverse background, resulting in the construction of more “realistic” micro-crack image data.

## 3. Robust Detection Model for Complex Scenes

Crack detection tasks still face three core challenges in feature modeling and information transmission:

1. Feature entanglement caused by environmental interference (lighting, shadows, stains, and crack texture similarity).
2. Difficulty in modeling long-range topological dependencies of cracks (elongated structures spanning large areas of pixels).
3. Degradation of small high-frequency features in deep networks (down-sampling and semantic aggregation lead to loss of details).

To address these issues, this paper proposes a robust detection model for complex scenes, named Complex-Scene-Tolerant YOLO (CST-YOLO). The model is built upon the YOLOv10 [24] framework, inheriting its efficient single-stage detection advantages while incorporating a design paradigm of “environment decoupling—global perception—small feature enhancement”. The network structure is specifically restructured and extended to better adapt to micro-crack detection tasks.

### 3.1. Overall Model Architecture

**Figure 2:**
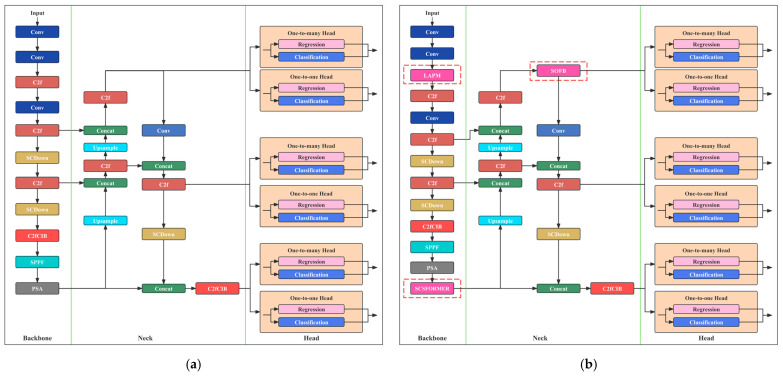
Comparison between the YOLOv10 Network Architecture and the CST-YOLO Network Architecture (Proposed Method). (**a**) The YOLOv10 Network Architecture; (**b**) The CST-YOLO Network Architecture (Proposed Method).

The overall network structure of CST-YOLO is shown in Figure 2b. The network can be divided into three hierarchical modules from input to output:

1. Input and lighting-adaptive preprocessing layer. Unlike standard YOLO pipelines that directly feed raw images into the network, CST-YOLO introduces an Lighting-Adaptive Preprocessing Module (LAPM) at the front end. Concretely, the input image is first processed by two Stem blocks to perform rapid down-sampling and obtain an initial feature representation. The resulting features are then forwarded to the LAPM, where feature-level illumination decoupling and noise suppression are conducted to attenuate the influence of lighting factors on crack feature extraction.
2. Multi-scale feature extraction backbone layer. The backbone adopts a CSP-based convolutional architecture to perform multi-scale feature extraction, generating feature maps at multiple resolutions via progressive down-sampling. Given that micro-cracks are highly sensitive to spatial resolution, CST-YOLO preserves relatively high-resolution shallow features at the backbone output, providing essential spatial detail to support subsequent small-target enhancement.
3. Feature fusion layer with global perception and micro-feature enhancement. To compensate for the limited ability of conventional CNNs to model long-range dependencies, CST-YOLO incorporates a Spatial-Channel Sparse Transformer (SCS-Former) at the end of the backbone to enhance understanding of the overall crack topology. By employing a sparsified attention mechanism, SCS-Former captures the spatial continuity of elongated cracks while keeping the computational overhead under control. To prevent micro-crack cues from being overwhelmed by dominant semantic information in deep layers, CST-YOLO introduces a Small Object Focus Block (SOFB) at the fusion stage. SOFB enhances high-frequency responses and performs channel recalibration, thereby reinforcing crack boundaries and fine-grained details. Together, SCS-Former and SOFB constitute the feature fusion module, enabling CST-YOLO to jointly leverage global structural awareness and local detail modeling.

This hierarchical design enables the network to focus on different tasks at each stage, such as lighting interference suppression, semantic feature extraction, structural relationship modeling, and refined object prediction, thus creating a clear division of functionality.

### 3.2. LAPM

#### 3.2.1. Theoretical Basis: Retinex-Based Image Decomposition Model

**Figure 3:**
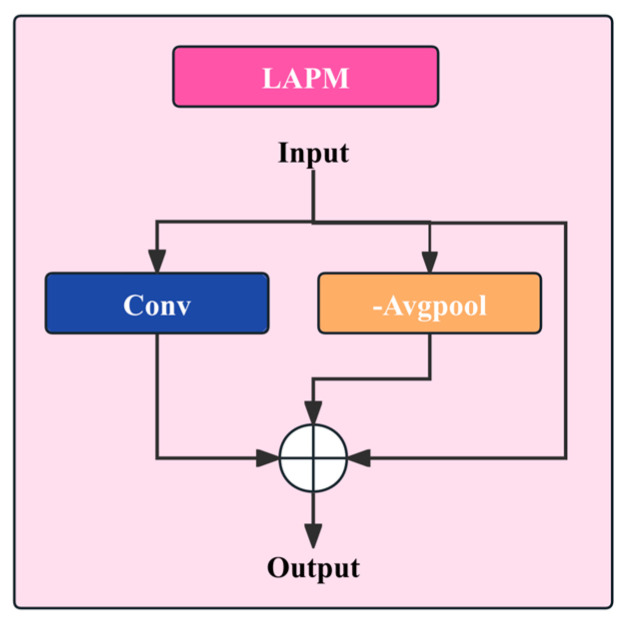
LAPM Network Architecture.

The design of the Lighting-Adaptive Preprocessing Module (LAPM) is motivated by the classical Retinex theory [36], and the network architecture is shown in Figure 3. Retinex postulates that human perception of a scene is primarily determined by the reflectance properties of object surfaces, whereas variations in illumination have a comparatively limited influence on the perceived appearance. In computer vision, Retinex is commonly formulated by decomposing an observed image into reflectance and illumination components, which can be expressed as follows:

(2) I=R⋅L

where I denotes the observed pixel intensity, R represents the reflectance component associated with intrinsic surface properties, and L denotes the illumination component induced by environmental lighting.

From a frequency-domain perspective, the illumination component typically varies smoothly across space and thus corresponds to a low-frequency signal, whereas the reflectance component contains abundant high-frequency information arising from textures, edges, and structural variations. For crack detection, cracks are structural defects on material surfaces, and their discriminative cues are mainly embedded in the reflectance-related high-frequency patterns. Therefore, suppressing illumination effects while enhancing reflectance-related responses is essential for improving crack separability under complex lighting conditions.

Nevertheless, conventional Retinex methods often rely on logarithmic transformations and multi-scale filtering, which are computationally demanding and difficult to integrate into deep neural networks in an end-to-end manner. To this end, while preserving the core Retinex rationale, we simplify and convolutionalize the modeling form so that it can be seamlessly embedded as a lightweight module within the detection network.

#### 3.2.2. Approximate Modeling and Estimation of the Illumination Component

In LAPM, the illumination component L is regarded as a low-frequency component of the image whose spatial variation is inherently smooth. Under this assumption, we apply a large-receptive-field average pooling operation, serving as a low-pass filter, to the input features to obtain an approximate estimate of the illumination term. Let the input feature map be $X\in R^{H\times W\times C}$, the illumination estimate is given by

(3) $$\hat{L}={AvgPool}_{k}(X)$$

where ${AvgPool}_{k}(\cdot )$ denotes the average pooling operator with kernel size k, and k=15 is used in this study. Compared with small-kernel convolutions, large-kernel average pooling aggregates information over a broader spatial neighborhood, thereby effectively suppressing local textures and high-frequency noise while preserving the global trend of intensity variation. Notably, this operation introduces no additional learnable parameters, making it simple to implement and stable in practice.

#### 3.2.3. Reflectance Component Extraction and Differential Enhancement Mechanism

After obtaining the illumination estimate, LAPM extracts an approximate reflectance component via a differencing operation to emphasize the structural characteristics of cracks. Specifically, the reflectance-related feature is defined as

(4) $$\hat{R}=X-\hat{L}$$

This differencing operation acts as a high-pass filter in the feature space. In regions where illumination varies smoothly, the difference tends to be close to zero, thereby suppressing background responses. In contrast, at locations with high-frequency structures, such as cracks, the pronounced discrepancies in intensity or texture between crack pixels and their surrounding background lead to strong responses in the difference map, thereby enhancing crack boundaries and skeleton-like cues. It is worth noting that this differencing formulation is not merely an edge detector; rather, it serves as a local-contrast-driven structural enhancement mechanism that preserves the integrity of crack morphology while attenuating large-scale illumination perturbations.

#### 3.2.4. Residual Fusion and Feature Stability Analysis

Although the reflectance component obtained by differencing can highlight crack-related cues, relying solely on it may weaken contextual information that is beneficial for discrimination, such as the global distribution of background textures or material attributes. To avoid excessive information loss, LAPM adopts a residual fusion strategy: a 3×3 convolution is first applied to further capture the original input features, and the result is then fused with the enhanced reflectance features as

(5) $$Y_{1}=Conv(X)+\hat{R}$$

This residual design theoretically improves the stability of feature mapping. On the one hand, high-frequency crack features are explicitly enhanced; on the other hand, preserving an original feature path prevents gradient instability during training that may arise from abrupt shifts in feature distributions. In addition, the residual connection facilitates faster convergence and improves overall training efficiency.

### 3.3. Spatial–Channel Sparse Transformer

#### 3.3.1. Design Rationale and Overall Framework

**Figure 4:**
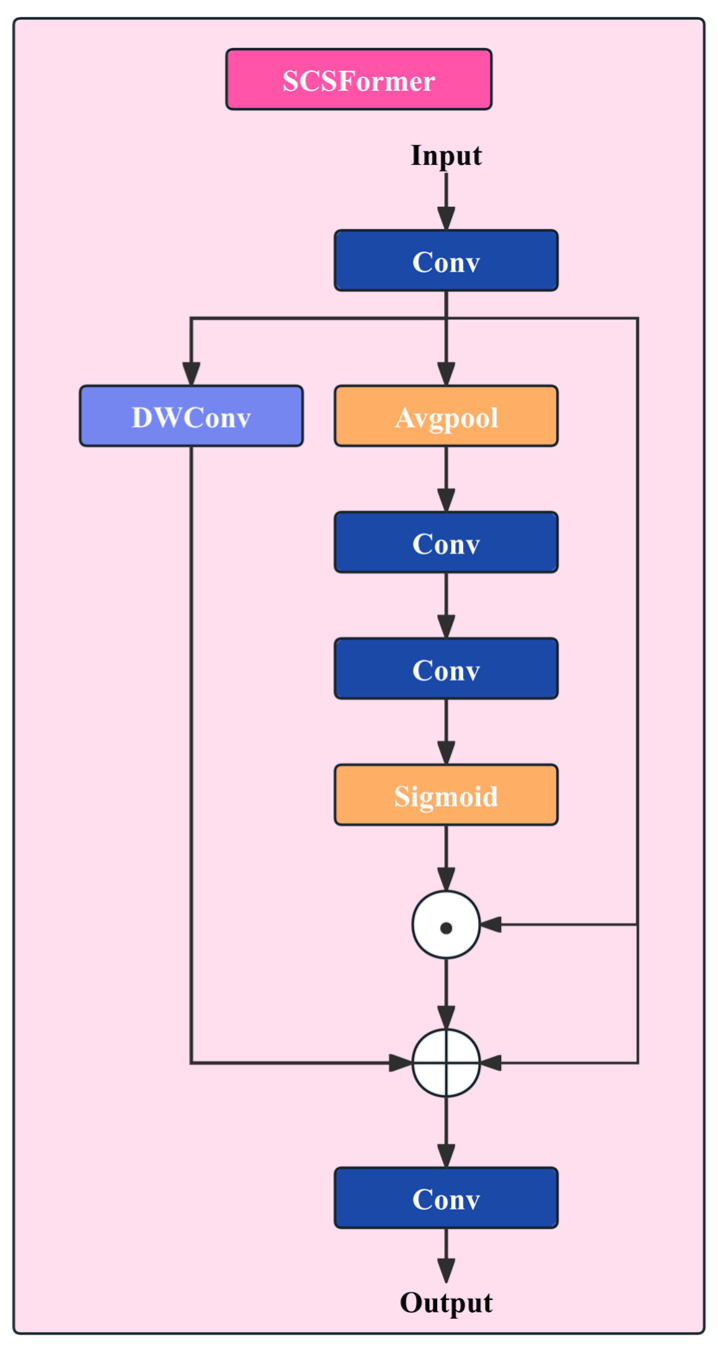
SCS-Former Network Architecture.

SCS-Former disentangles highly coupled spatial–channel attention in conventional Transformers by modeling global dependencies separately along the spatial and channel dimensions; the network architecture is shown in Figure 4. Specifically, the input features are first linearly projected to obtain a unified representation, which is then fed into a spatially sparse branch and a channel attention branch, followed by a final fusion operation. This “divide-and-conquer” decoupling strategy allows the model to focus on the continuity and geometry of crack patterns in the spatial domain, while selecting channel-wise feature subspaces that are more sensitive to crack semantics, thereby improving discriminative stability under complex backgrounds.

#### 3.3.2. Spatial Sparse Attention Modeling

In the spatial branch, SCS-Former employs a large-kernel depth-wise convolution (DWConv) to emulate a sparse attention mechanism along the spatial dimension. Compared with standard convolutions, small kernels can only capture local neighborhood information, whereas large kernels substantially expand the effective receptive field. This enables the model to acquire cross-region spatial context without explicitly constructing a global attention matrix of size HW×HW as in conventional Transformers. In this branch, a 7×7 kernel is adopted instead of the commonly used 3×3 kernel, providing a larger physical receptive field. In crack detection scenarios, cracks typically appear as thin elongated patterns spanning a wide area; a limited receptive field may fragment such structures into seemingly unrelated responses, whereas a 7×7 window can cover longer crack segments and thus better capture their continuity.

Specifically, the spatial sparse attention is formulated as

(6) $$X_{s}={DWConv}_{7\times 7}(X^{\prime })$$

This operation performs spatial aggregation independently within each channel, which is equivalent to imposing structured spatial attention weights on each feature channel. Such a design introduces a strong spatial inductive bias, guiding the model to focus on the spatial continuity of cracks while suppressing global interference from irrelevant regions.

#### 3.3.3. Channel Attention Modeling

One major challenge in crack detection is that background noise—such as shadows, water stains, and pavement granules—can be highly confusable with real cracks in terms of texture. Certain feature channels may be sensitive to cracks, whereas others may respond more strongly to noise. Therefore, a mechanism is required to dynamically select the “important” channels.

In the channel branch, SCS-Former employs Global Average Pooling (GAP) to extract global statistics per channel. For the projected feature map $X^{\prime }$, the channel descriptor vector $z_{c}\in R^{C}$ is defined as

(7) $$z_{c}=\frac{1}{HW}\sum_{i=1}^{H}\;\sum_{j=1}^{W}\;X^{\prime }(i,j,c)$$

This global descriptor compresses spatial information into the channel dimension, enabling the model to explicitly characterize the global importance of different feature channels. Subsequently, the channel descriptor is fed into a mapping function composed of two 1×1 convolutions and a nonlinear activation to generate channel attention weights:

(8) $$w=\sigma (\psi (z_{c}))$$

where σ(⋅) denotes the Sigmoid activation function and ψ(⋅) represents the convolutional transformation.

Finally, channel-wise attention weights are applied to the input features:

(9) $$X_{c}=w⊙X^{\prime }$$

#### 3.3.4. Spatial–Channel Feature Fusion Mechanism

After obtaining the spatial sparse feature $X_{s}$ and the channel-enhanced feature $X_{c}$, SCS-Former fuses the outputs of the two branches with the input feature via element-wise addition, and then applies a 1×1 convolution to produce the output feature:

(10) $$Y_{2}=Conv(X_{s}+X_{c}+{\;X}^{\prime })$$

This fusion strategy is structurally analogous to a residual connection. Its key idea is to form a lightweight feature pyramid that enables multi-level feature complementarity: the original feature branch preserves input details to prevent the loss of fundamental information; the spatial sparse branch captures long-range spatial dependencies of cracks via large-kernel depth-wise convolution, thereby modeling structural continuity; and the channel attention branch dynamically recalibrates feature channels to enhance the discrimination between cracks and background. By summing these components, the module efficiently integrates details, context, and saliency, avoiding the dimensional expansion introduced by feature concatenation while maintaining stable gradient propagation via the residual-style connection.

Compared with standard Transformers, SCS-Former avoids constructing an attention matrix of size HW×HW. Its computational cost is mainly dominated by large-kernel depth-wise convolutions and channel-mapping operations, leading to an approximately linear growth in overall complexity. This property enables SCS-Former to maintain favorable computational efficiency for high-resolution crack-detection tasks.

Moreover, SCS-Former explicitly introduces inductive biases tailored to crack morphology in its structural design: spatial sparse attention strengthens the long-range continuity of cracks, while channel attention suppresses channels dominated by background noise. Such structured attention is more consistent with the physical and geometric characteristics of crack detection than unconstrained global self-attention.

### 3.4. Small Object Focus Module

#### 3.4.1. Learnable Second-Order Differential High-Frequency Feature Enhancement

**Figure 5:**
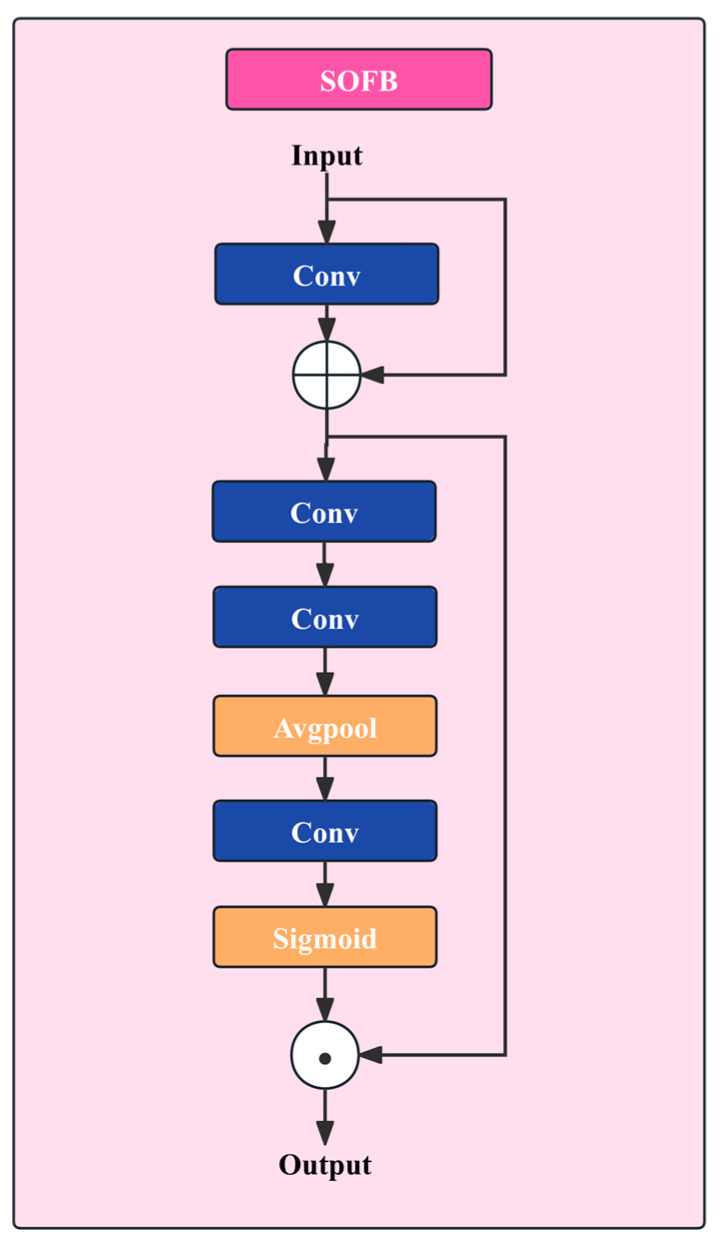
SOFB Network Architecture.

From a signal-processing perspective, cracks essentially correspond to regions with abrupt intensity variations in an image, and thus belong to the high-frequency components. During the layer-wise stacking process, conventional convolutions tend to favor low-frequency semantic information, which gradually weakens edge and fine-detail features. To address this issue, SOFB introduces a high-frequency feature enhancement unit at the module front-end, inspired by second-order differential operators, to explicitly amplify crack-edge responses. The network architecture is shown in Figure 5.

The Laplacian operator is an isotropic second-order differential operator that is highly sensitive to sharp intensity changes in images. For a two-dimensional image function f(x,y), its Laplacian transform is defined as

(11) $$\Delta^{2}f=\frac{{𝜕}^{2}f}{𝜕x^{2}}+\frac{{𝜕}^{2}f}{𝜕y^{2}}$$

In discrete concrete image data, this operator is typically implemented via a convolution kernel. By computing differences between the central pixel and its neighboring pixels, it can effectively extract crack edge and corner information. A typical 3×3 Laplacian kernel $K_{\Delta }$ is defined as

(12) $$K_{\Delta }=\left[\begin{array}{ccc} 0 & -1 & 0\\ -1 & 4 & -1\\ 0 & -1 & 0 \end{array}\right]$$

In SOFB, the high-frequency enhancement convolution layer is initialized with weights in the form of $K_{\Delta }$, while allowing the parameters to be adaptively updated during training. This “prior initialization + data-driven fine-tuning” scheme endows the network with edge sensitivity early in training and enables it to adapt to different scenes.

#### 3.4.2. High-Frequency Enhancement and Residual Fidelity Mechanism

Although fixed high-pass filtering can highlight edge information, directly replacing the original features with such outputs may severely discard low-frequency contextual cues, thereby weakening the model’s understanding of the overall crack semantics. To address this issue, SOFB adopts a residual connection to fuse the high-frequency enhanced features with the original input features, formulated as

(13) $$X_{\mathrm{enh}}=X+H(X)$$

where X denotes the input feature map and H(⋅) represents the high-frequency enhancement convolution operation. This residual design strengthens crack edges while preserving contextual information in the original features, enabling a better balance between detail amplification and semantic fidelity. Moreover, the residual path facilitates stable gradient propagation and helps prevent numerical instability during training.

#### 3.4.3. Batch-Normalization-Free Channel Attention Mechanism

Micro-crack features are extremely sparse in spatial distribution, and their statistical properties differ substantially from those of large background regions. Conventional channel attention modules, such as the SE block, typically rely on batch normalization (BN) to standardize feature distributions. However, when the proportion of small targets is extremely low, the mean and variance estimated by BN are often dominated by background noise, making it difficult to accurately reflect the true distribution of crack-related features.

To avoid this issue, SOFB explicitly removes the BN layers from the channel-attention branch and models channel-wise weights solely from the raw feature responses. Specifically, after two convolutional operations are applied to further extract spatial and channel features, global average pooling is first used to compress the spatial dimension, producing a channel descriptor vector:

(14) $$z_{c}=\frac{1}{HW}\sum_{i=1}^{H}\;\sum_{j=1}^{W}\;X_{enh}(i,j,c)$$

Then, the channel descriptor is mapped to channel weights through a lightweight mapping network composed of a 1×1 convolution and a nonlinear activation function:

(15) $$w=\sigma (\psi (z_{c}))$$

where σ(⋅) denotes the Sigmoid activation function and ψ(⋅) represents the convolutional transformation.

Finally, the channel weights are applied to the enhanced features in a channel-wise manner to strengthen crack-sensitive channels while suppressing channels dominated by background noise:

(16) $$Y_{3}=w⊙X_{enh}$$

The design of SOFB explicitly considers statistical stability in micro-crack detection. By removing BN and adopting a global-statistics-based channel recalibration mechanism, the module mitigates the disturbance caused by variations in crack proportions across mini-batches, thereby improving training stability. Meanwhile, the synergy between high-frequency enhancement and channel attention enables the model to focus more reliably on crack-related cues under complex backgrounds, significantly reducing false detections induced by background texture interference.

## 4. Experimental Design and Result Analysis

### 4.1. Experimental Setup

To comprehensively validate the effectiveness and robustness of the proposed CST-YOLO model for micro-crack detection in complex scenes, we conduct systematic comparative experiments and ablation studies under a unified experimental environment and consistent data settings. This section describes the experimental setup in detail, including the hardware platform and software configuration, to ensure the reproducibility and fairness of the reported results.

#### 4.1.1. Hardware and Software Environment

All experiments in this study were conducted under the same hardware and software environment to eliminate the influence of computational platform variations on the experimental results. The hardware and software configurations used in the experiments are summarized in Table 2.

During training, all models were trained on a single GPU and initialized with the same random seed to ensure comparability across different experiments.

#### 4.1.2. Training Configuration Parameter Settings and Evaluation Metrics

To ensure fair training and reproducible results across all comparative methods, we used the AdamW optimizer for end-to-end parameter optimization. The input resolution was set to 640×640, with an initial learning rate of $1\times 10^{-2}$ and a weight decay coefficient of $5\times 10^{-4}$. All models were trained for 300 epochs with a batch size of 32. Except for architectural differences, all other training settings were kept identical across methods. A polynomial (Poly) learning-rate schedule was used with the power parameter p=0.9. Each model was trained until convergence, and the checkpoint achieving the best validation performance was selected for subsequent testing. In addition, we adopted the overall loss function of YOLOv10 for end-to-end training.

Given that crack detection typically involves small, elongated targets and that missed detections can be particularly costly, we adopted four evaluation metrics—Precision, Recall, mAP@0.5, and mAP@0.5:0.95—to comprehensively assess model performance from multiple perspectives.

### 4.2. Dataset Construction and Composition

We constructed a crack dataset comprising 2000 images: 700 real crack images and 1300 synthetic crack images generated via Poisson editing. In addition, cracks with different aspect ratios were selected, with aspect ratios ranging from 5:1 to 30:1. The real data were collected from high-resolution images of concrete surfaces captured in practical engineering scenarios, covering multiple types of infrastructure, such as bridges and roads. These images exhibit complex illumination conditions, diverse background textures, and multi-scale crack morphologies.

Given the pronounced imbalance in both the quantity and scale distributions of real crack samples, the Poisson editing-based crack synthesis method described in Section 3.2 was incorporated into the training procedure to supplement and expand the training data.

The synthetic data were used only for model training. Both the validation set and the test set were constructed exclusively from real crack images to avoid potential biases introduced by synthetic samples and to ensure a faithful evaluation of real-world performance. To ensure objective and stable experimental results, the dataset was split at the image level, preventing different tiles cropped from the same original image from appearing simultaneously in the training and test sets. The training, validation, and test sets were mutually exclusive, with a 7:2:1 split.

### 4.3. Overall Performance Evaluation of CST-YOLO

In this section, we evaluate the overall performance of CST-YOLO for micro-crack detection in complex scenes by conducting comparative experiments against several mainstream object detection methods. Both quantitative metrics and qualitative visualizations are provided. All experiments follow the unified experimental settings described in Section 4.1 and adopt the evaluation metrics defined in Section 4.2.

To comprehensively and objectively assess the performance advantages of CST-YOLO in complex-scene micro-crack detection, we select representative detectors from different categories as baselines and perform both quantitative and qualitative analyses using multi-dimensional evaluation metrics. To avoid one-sided comparisons, the following methods are included:

1. Two-stage detectors typically prioritize accuracy at the expense of lower inference efficiency. We choose Faster R-CNN [20] as a representative two-stage method, using ResNet-18 [37] as the backbone.
2. One-stage detectors are the mainstream paradigm that balances speed and accuracy. We include SSD-Lite [38] as a representative lightweight detector, along with several YOLO-family baselines, including YOLOF [23], YOLOX [22], and YOLOv12 [25]. These methods have been widely adopted in both academia and industry and reflect the current mainstream performance of one-stage detectors in terms of efficiency and accuracy.
3. Transformer-based [39] detection frameworks represent an emerging paradigm that redesigns the detection pipeline via attention mechanisms. We introduce RT-DETR-R50 [26] as a baseline, utilizing pre-trained models on the COCO dataset. It performs global feature interaction and bounding-box prediction in a single pass via an encoder–decoder architecture, without requiring NMS post-processing, thereby enabling near-real-time inference while maintaining high accuracy. This provides a direct reference for comparing CNN-based and Transformer-based detectors in the object detection setting.

Table 3 reports the quantitative results of CST-YOLO and all baselines on the test set, including Precision, Recall, mAP@0.5, mAP@0.5:0.95, and inference efficiency metrics.

From the quantitative comparisons in Table 3, the proposed CST-YOLO achieves a well-balanced trade-off between detection accuracy and inference efficiency for micro-crack detection, demonstrating the most competitive overall performance. Specifically, CST-YOLO achieves a Precision of 0.979 and a Recall of 0.991, indicating a low false-positive rate while maintaining high crack detection completeness. Moreover, CST-YOLO achieves the highest mAP@0.5:0.95 score of 0.926, substantially outperforming Faster R-CNN and YOLOv12. This result suggests that CST-YOLO preserves stable detection and localization capability under a stricter set of IoU thresholds, which is particularly critical for micro-cracks characterized by thin, elongated shapes, low contrast, and blurred boundaries. Notably, although Faster R-CNN benefits from its two-stage architecture and reaches 1.000 on both mAP@0.5 and Recall, its performance drops markedly on mAP@0.5:0.95. This indicates insufficient localization refinement under high IoU thresholds and/or unstable boundary fitting in crack structures. YOLOv12 yields relatively high Precision and mAP@0.5; however, it remains inferior to CST-YOLO under more stringent multi-threshold evaluation, implying that CST-YOLO offers stronger generalization and robustness across varying IoU requirements. In terms of efficiency, CST-YOLO achieves an inference speed of 139 FPS, which is close to the fastest model (YOLOv12) and significantly higher than the other baselines, highlighting its potential for real-time deployment in practical engineering applications. In terms of efficiency, CST-YOLO achieves an inference speed of 139 FPS with 2.74 M parameter counts and 8.90 G FLOPs, approaching the fastest model (YOLOv12). Its inference efficiency is significantly higher than that of other benchmark models, highlighting its potential for real-time deployment in practical engineering applications. By contrast, RT-DETR-ResNet50 exhibits notably lower accuracy across metrics. This is likely attributable to the limited training data available in this study (only 1400 images), suggesting that Transformer/DETR-style detectors may be more sensitive to data scale and training stability and therefore struggle to learn fine-grained crack morphology and texture distributions under small-sample settings. Overall, the experimental results validate that CST-YOLO provides superior comprehensive performance for micro-crack detection with limited data, achieving leading results on the strict mAP@0.5:0.95 metric while maintaining high inference efficiency, thus offering a more reliable choice for subsequent engineering deployment.

**Figure 6:**
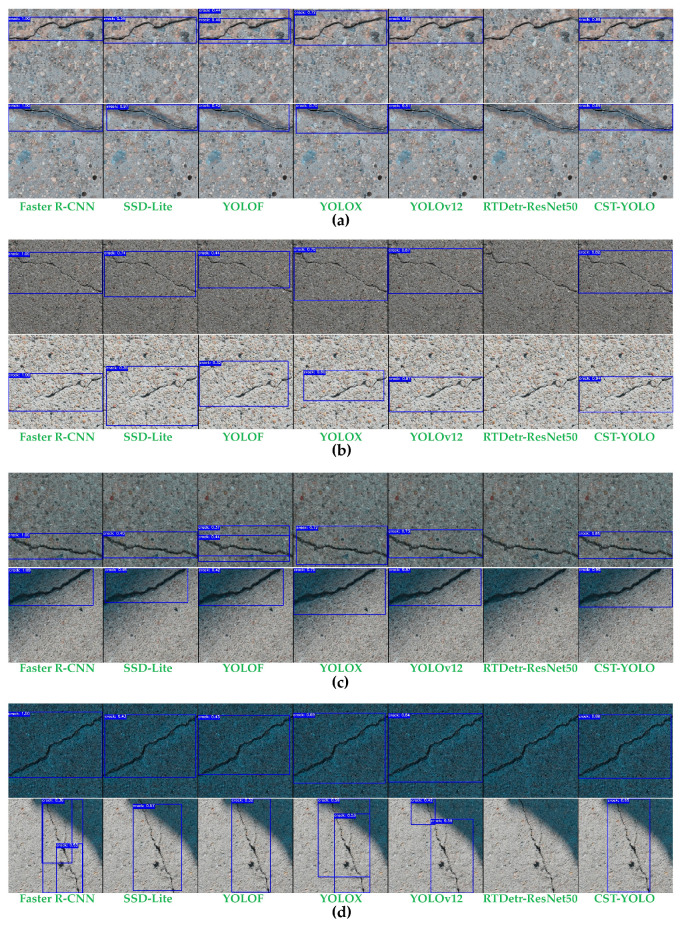
Detection results generated by different models on a subset of images from the test set: (**a**) Image group with strong background noise; (**b**) image group with very fine cracks; (**c**) image group under low-illumination conditions; (**d**) image group under non-uniform illumination conditions.

Figure 6 presents detection results produced by different models on eight images selected from the test set, which are loosely categorized into four groups for analysis. Group (a) contains images with strong background noise, where both samples exhibit pronounced texture patterns. Group (b) includes images with very thin cracks; under strong illumination, the contrast between the cracks and the background is low. Group (c) comprises low-illumination images, and the second sample also exhibits pronounced background texture noise. Group (d) consists of images under non-uniform illumination: the first sample shows a clear shadow boundary, while the second can be regarded as being captured in a dim-light environment.

These visualizations indicate that RT-DETR-ResNet50 fails to provide reliable detection with a limited dataset. Compared with SSD-Lite, YOLOF, and YOLOX, Faster R-CNN and YOLOv12 achieve higher precision in most scenarios; however, relative to CST-YOLO, both methods show insufficient understanding of the global morphology of thin cracks and the associated boundary context, leading to degraded localization accuracy and redundant detections (e.g., the images in group (b)). In addition, the receptive field of deep features remains insufficient to capture the full extent of long cracks, leading to the misclassification of local contextual patterns as crack segments. Their inference efficiency is also lower, which makes it difficult to satisfy the speed requirements in practical engineering applications (e.g., the second image in group (d)). In contrast, CST-YOLO achieves a more favorable balance among precision, recall, and efficiency.

Based on the qualitative results, CST-YOLO exhibits clear advantages in maintaining crack continuity, filling fragmented regions, and suppressing background interference. Several baseline methods suffer from missed detections or false positives in areas with extremely thin cracks or severe illumination variations, whereas CST-YOLO detects crack targets more completely and maintains spatially coherent bounding-box predictions.

### 4.4. Ablation Studies and Module Effectiveness Analysis

#### 4.4.1. Ablation Studies of Each Module of CST-YOLO

To further investigate the individual contributions of each CST-YOLO module and their synergistic effects on micro-crack detection in complex scenes, we conducted a series of ablation studies under the unified experimental settings. By progressively introducing key components into the baseline model, we systematically analyze how each module affects detection performance, thereby substantiating the effectiveness and advantages of the proposed design.

To ensure a fair comparison, all networks were trained using the same dataset and identical hyperparameter configurations. For each variant, we recorded the curves of the evaluation metric over epochs. In total, six experimental settings were considered:

- The YOLOv10 baseline, shown as the blue curve in the following figures;
- YOLOv10_LAPM (The YOLOv10 baseline augmented with the LAPM), shown as the orange curve;
- YOLOv10_SCS-Former (The YOLOv10 baseline augmented with SCS-Former), shown as the green curve;
- YOLOv10_SOFB (The YOLOv10 baseline augmented with the SOFB), shown as the red curve;
- YOLOv10_EAMP_SCS-Former (The YOLOv10 baseline augmented with both LAPM and SCS-Former), shown as the purple curve;
- The full CST-YOLO model is shown as the brown curve.

**Figure 7:**
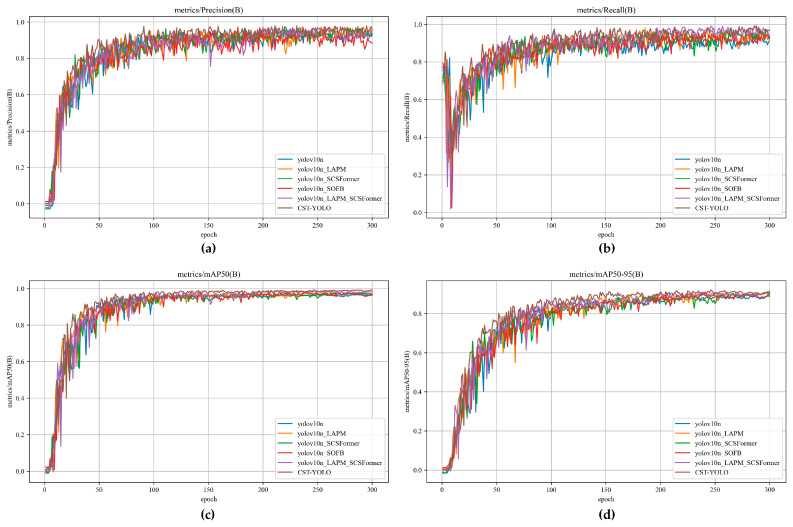
The Comparison of Evaluation Metric Curves between Different Modules. (**a**) Precision; (**b**) Recall; (**c**) mAP@0.5; (**d**) mAP@0.5:0.95.

The comparison of evaluation metrics across modules is presented in Figure 7 and Table 4.

The results show that introducing LAPM, SCS-Former, and SOFB individually leads to noticeable improvements in Precision, Recall, mAP@0.5, and mAP@0.5:0.95, while the computational efficiency does not exhibit a significant decline. When LAPM, SCS-Former, or SOFB is incorporated separately, inference time per image increases only slightly; precision and Recall are largely unaffected, whereas mAP@0.5 and mAP@0.5:0.95 improve to varying extents. After progressively integrating all three modules, the performance is further enhanced, reaching 0.979 in Precision, 0.991 in Recall, 0.992 in mAP@0.5, and 0.926 in mAP@0.5:0.95. Overall, these results indicate that CST-YOLO achieves higher accuracy in crack detection, reducing both missed detections and false positives.

**Figure 8:**
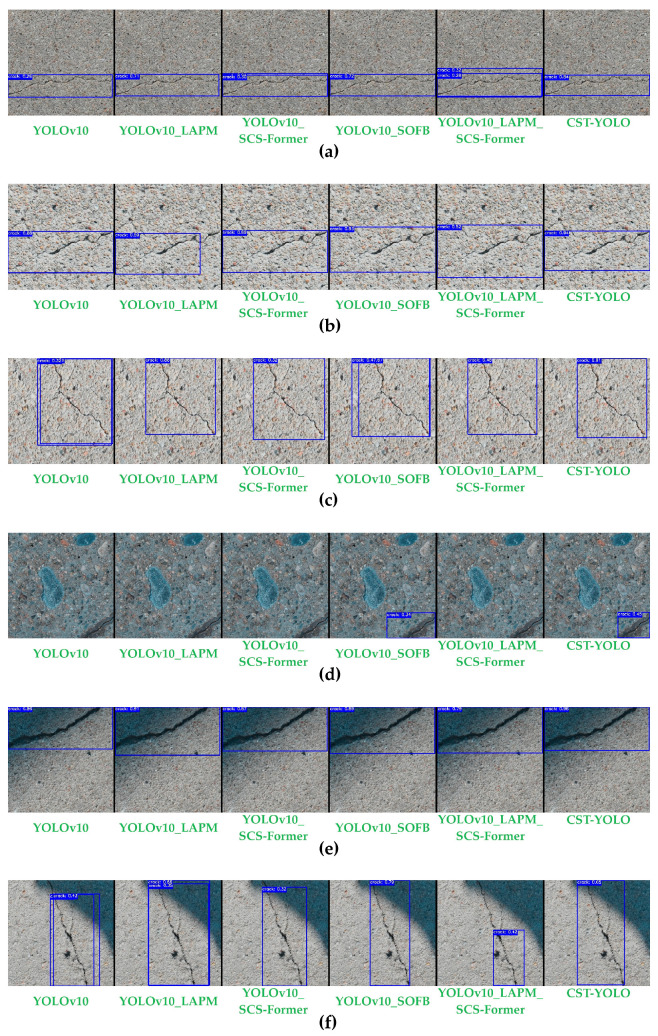
Ablation Results on Images. (**a**) Long-distance Micro-cracks; (**b**) Micro-cracks under High-lighting Conditions; (**c**) Long-distance Micro-cracks under High-lighting Conditions; (**d**) Micro-cracks; (**e**) Cracks in Varying lighting Environments; (**f**) Long-distance Micro-cracks in Varying lighting Environments.

Figure 8 presents qualitative ablation results on six representative images. As shown in Figure 8e,f, after introducing the LAPM, the model’s ability to detect cracks on surfaces with non-uniform illumination and shadowed backgrounds is substantially improved, with fewer missed detections. This indicates that by decoupling the illumination component from the structural component at the network front-end, LAPM effectively alleviates the adverse impact of lighting interference on crack feature extraction, thereby providing more stable input features for subsequent modules.

In Figure 8f, after incorporating the SCS-Former module, the model exhibits a markedly enhanced capability to capture long-range crack structures. Long cracks can be detected more completely, and missed detections at crack endpoints are mitigated. This suggests that the spatially sparse attention mechanism introduced by SCS-Former plays a key role in capturing the global structural information of cracks.

For Figure 8a–d, the full model further improves micro-crack detection beyond the aforementioned enhancements. In particular, cracks with widths of only a few pixels are less likely to be missed, and the increased confidence in the predicted bounding boxes also indicates greater reliability in detecting cracks in complex scenes. These observations demonstrate that the high-frequency feature enhancement and channel recalibration mechanisms introduced by SOFB effectively strengthen micro-crack responses in deep features, serving as an important factor in improving small-target detection capability.

Overall, the ablation results indicate that the modules in CST-YOLO improve performance in a complementary manner across different levels: LAPM suppresses lighting interference, SCS-Former models the global crack structure, and SOFB amplifies micro-scale high-frequency features, thereby forming a clear functional division of labor. The results confirm that each module plays an indispensable role in improving micro-crack detection accuracy, reducing missed detections, and enhancing robustness under complex scenes, thereby further validating the rationale of the proposed model design.

#### 4.4.2. Ablation Studies on Poisson Image Editing-Based Data Fusion

To further evaluate the contribution of the proposed Poisson image editing-based data fusion strategy, an ablation study was conducted under the same experimental settings by comparing CST-YOLO-With-PoissonBlending and CST-YOLO-Without-PoissonBlending. Except for the data construction strategy, all experimental conditions, including the detector architecture, optimization settings, training epochs, and evaluation criteria, were kept the same to ensure a fair comparison. Specifically, the dataset used by CST-YOLO-Without-PoissonBlending consisted of the 700 real images described in Section 4.2, which were divided into the training, validation, and test sets at a ratio of 7:2:1. CST-YOLO-With-PoissonBlending introduced the proposed Poisson image editing-based dataset in Section 4.2 during sample construction. Therefore, the effect of Poisson blending can be isolated from other factors and quantitatively assessed.

As listed in Table 5, the introduction of Poisson blending leads to consistent improvements across all evaluation metrics. Specifically, CST-YOLO-With-PoissonBlending achieved a Precision of 0.989, a Recall of 0.997, an mAP@0.5 of 0.995, and an mAP@0.5:0.95 of 0.938, whereas CST-YOLO-Without-PoissonBlending obtained 0.979, 0.991, 0.992, and 0.922, respectively. Compared with the model trained without Poisson blending, the proposed strategy improved Precision by 1.03 percentage points, Recall by 0.57 percentage points, mAP@0.5 by 0.24 percentage points, and mAP@0.5:0.95 by 1.65 percentage points. Although the gain in mAP@0.5 is relatively limited because both settings already reached a high detection level, the more noticeable improvement in mAP@0.5:0.95 indicates that Poisson blending contributes not only to crack recognition but also to more accurate localization under stricter IoU thresholds. This improvement is particularly meaningful for micro-crack detection, since such targets usually exhibit extremely slender morphology, weak contrast, and ambiguous boundaries.

**Figure 9:**
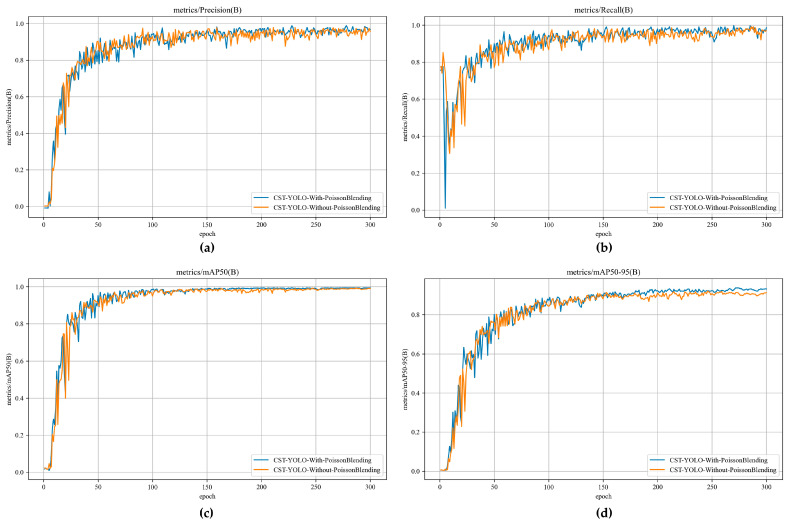
The Comparison of Evaluation Metric Curves between Ablation Studies on Poisson Image Editing-Based Data Fusion. (**a**) Precision; (**b**) Recall; (**c**) mAP@0.5; (**d**) mAP@0.5:0.95.

The convergence curves provide further evidence for the effectiveness of the proposed strategy. As shown in Figure 9, the CST-YOLO-with-PoissonBlending is shown as the blue curve and the CST-YOLO-Without-PoissonBlending is shown as the orange curve. The mAP@0.5 curves of the two settings both increase rapidly in the early stage and gradually converge after sufficient training; however, the model trained with Poisson blending maintains a slightly higher performance level over most epochs. A similar trend can be observed in Figure 9d for mAP@0.5:0.95, where the advantage of CST-YOLO-With-PoissonBlending becomes more evident in the middle and later training stages. This suggests that Poisson image editing not only improves the convergence behavior of the detector, but also raises the upper bound of detection performance under more stringent localization requirements.

As shown in Figure 9a,b, the Precision and Recall curves also support the superiority of the proposed strategy. Although both models eventually achieve relatively high values, CST-YOLO-With-PoissonBlending exhibits a better overall balance between precision and recall during training. In particular, the model with Poisson blending maintains a slightly higher and more stable recall in the later epochs, indicating that more true crack instances can be preserved under complex background interference. Meanwhile, its precision remains consistently competitive, which implies that the increase in recall is not obtained at the expense of substantially more false positives. For crack detection tasks, this balance is important because missed detections of weak and discontinuous crack segments may directly affect the completeness of structural damage assessment.

The above improvements can be attributed to the intrinsic advantage of Poisson image editing in gradient-domain fusion. Unlike direct copy-paste or conventional image stitching methods, Poisson blending can better preserve boundary continuity and grayscale transition consistency between the crack foreground and the concrete background. Consequently, it alleviates visual artifacts such as edge discontinuities, abrupt intensity changes, and unrealistic pasted traces in synthesized images. As a result, the generated crack samples are more visually natural and more similar to real inspection images. This enables the detector to focus more on intrinsic crack characteristics, such as elongated topology, weak texture response, and subtle local structural variations, rather than overfitting to artificial fusion patterns introduced during data construction. Therefore, the detector trained with Poisson-blended samples exhibits stronger robustness and better localization capability when applied to real crack scenes.

Overall, the ablation results demonstrate that the proposed Poisson image editing-based data fusion strategy is an effective component of the entire framework. Without changing the detector architecture, the introduction of Poisson blending consistently improves Precision, Recall, mAP@0.5, and especially mAP@0.5:0.95 on the basis of the same real-image dataset. These results indicate that the role of Poisson blending lies not merely in increasing the quantity of training samples, but more importantly in enhancing the realism, distribution compatibility, and task relevance of synthetic crack data. Therefore, the proposed strategy provides effective data-level support for improving the detection performance of CST-YOLO in complex concrete crack scenarios.

## 5. Conclusions

This paper addresses micro-crack detection in concrete images under complex real-world conditions by jointly improving training data realism and feature modeling robustness. On the data side, a Poisson image editing-based synthesis strategy is proposed to generate realistic micro-crack samples with coherent texture and illumination transitions, easing the burden of limited labeled data. On the model side, we develop CST-YOLO on top of YOLOv10 and organize the architecture around the “lighting decoupling–global perception–micro-feature enhancement” architecture. LAPM stabilizes early feature extraction by reducing illumination and shadow interference, SCS-Former strengthens long-range structural modeling for elongated and discontinuous cracks with efficient sparse attention, and SOFB enhances micro-scale cues while suppressing background noise through feature focusing and channel recalibration.

Experiments on a 2000-image dataset (700 real images and 1300 synthesized images used only for training) show that CST-YOLO achieves 0.992 mAP@0.5 and 0.926 mAP@0.5:0.95 while running at 139 FPS, indicating reliable localization under strict IoU thresholds and practical real-time potential. Ablation results verify that the Poisson Image Editing-Based Data Fusion each proposed module contributes measurable improvements and that their combination delivers the strongest overall performance, particularly for thin cracks under non-uniform illumination and shadowed backgrounds. Future work will focus on expanding real-world data coverage across more structures and acquisition conditions, and on strengthening cross-domain robustness to ensure reliable deployment under broader variations in texture, lighting extremes, and imaging devices.

## 6. Discussion

Despite the impressive performance of CST-YOLO, several failure cases highlight its limitations in challenging scenarios, particularly with respect to thin cracks, complex backgrounds, and environmental disturbances. The following analysis considers detection results across various models, focusing on scenarios where CST-YOLO, along with other mainstream detection models, struggles.

**Figure 10:**
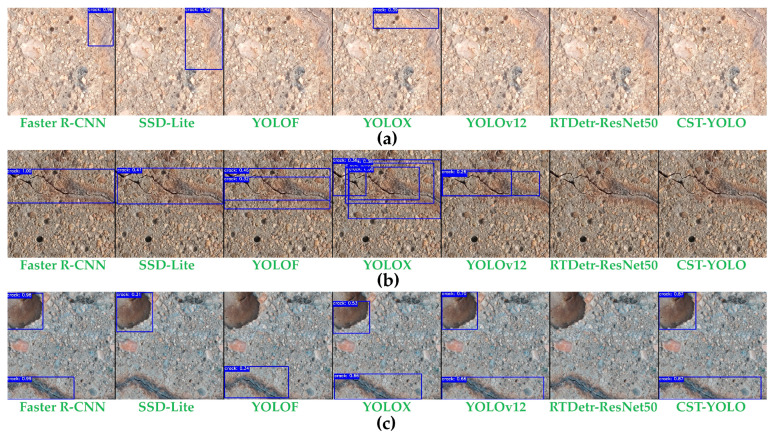
Failure cases. (**a**) Case 1: Detection of Thin Cracks; (**b**) Case 2: Complex Background Interference; (**c**) Case 3: False Positives in Crack-Like Texture Regions.

**Case 1: Detection of Thin Cracks.** The first image shows a failure to detect extremely thin cracks, which are challenging for all models. CST-YOLO, while exhibiting high precision in most cases, fails to adequately capture the faint details in such cracks. The output from CST-YOLO, like YOLOF and SSD-Lite, tends to miss these small cracks, leading to a noticeable drop in recall. Compared to Faster R-CNN and YOLOv12, CST-YOLO’s recall for thin cracks is lower, which is evident from the gaps in the bounding boxes in the visual results (Figure 10a). While YOLOv12 shows slightly better recall, it still fails in terms of boundary refinement, a critical aspect of fine-scale crack detection. These results point to the challenge of modeling fine-grained details in low-contrast areas where the crack width is minimal.

**Case 2: Complex Background Interference.** In scenarios with complex backgrounds (Figure 10b), such as cracks obscured by stains or shadows, CST-YOLO’s performance is impacted, though it outperforms the other models, especially RTDetr-ResNet50. CST-YOLO incorporates advanced modules like the Small Object Focus Block (SOFB) to enhance fine details, but in the presence of strong noise, it sometimes struggles with background separation. The bounding boxes from Faster R-CNN, YOLOF, and SSD-Lite, although precise in some regions, frequently fail to delineate cracks clearly in these cluttered environments. YOLOv12 shows high precision but misses fine details in noisy regions, while RTDetr-ResNet50 exhibits a high false negative rate due to its sensitivity to background interference and the limited dataset.

**Case 3: False Positives in Crack-Like Texture Regions**. Figure 10c illustrates how CST-YOLO and the other compared models produce false positives in the presence of strong background textures. In such cases, the background contains complex texture patterns that can be easily misclassified as cracks. The performance of CST-YOLO is broadly similar to that of the other models. Although it has been optimized for small-object detection, false detections still occur under high-noise background conditions. In regions where the visual appearance of cracks is highly similar to that of background textures, CST-YOLO tends to mistakenly identify these non-crack regions as cracks, thereby reducing detection precision. This issue becomes especially pronounced in complex background scenes. Although the SOFB module enhances fine-grained features of small objects, CST-YOLO remains prone to misclassification when dealing with regions with high crack–background texture similarity.

Although CST-YOLO demonstrates strong performance in many aspects, particularly in small-object detection and multi-scale feature enhancement, it still exhibits certain limitations when dealing with extremely fine cracks, complex backgrounds, and challenging illumination conditions. These failure cases highlight that micro-crack detection remains a difficult task, especially in real-world scenarios characterized by diverse environmental conditions and varying lighting patterns, where a small number of missed detections and false positives still occur. These results further indicate the necessity of continued architectural refinement, potentially through more effective preprocessing techniques or stronger multi-scale detection strategies, in order to better handle such extreme cases. Future work may focus on further improving background noise suppression, enhancing detection performance in regions with uneven illumination, and strengthening the discrimination capability between cracks and complex background textures.

In the future word, although CST-YOLO achieves favorable accuracy and efficiency for micro-crack detection, several limitations remain. First, the current framework is deterministic and does not explicitly address annotation noise or predictive uncertainty. This is particularly important for micro-crack inspection, because crack traces are often only a few pixels wide and their boundaries can be inherently ambiguous under uneven illumination, stains, shadows, and low-contrast backgrounds. In addition, manual labeling of such fine structures is inevitably affected by subjective judgment, which may introduce boundary inconsistency and noisy annotations. Second, although this study identifies cross-scenario generalization as an important challenge, the present Poisson-based synthesis strategy is mainly used for augmentation, and CST-YOLO itself does not yet include an explicit domain adaptation mechanism. Therefore, discrepancies between augmented distributions and real-world crack distributions may still limit robustness under unseen domains. Recent studies indicate that these issues may be alleviated through uncertainty-aware learning and unsupervised domain adaptation. For example, SelectSeg improves reliability under limited-data and noisy-label conditions by combining uncertainty estimation, noisy-annotation filtering, semi-supervised retraining, and selective prediction [40], while STDASeg and FMU-UDA show that image blending, Poisson-based mixing [41], Fourier-Morphology blending, and self-training can effectively reduce source–target discrepancy in crack analysis [42].

Another limitation is that the proposed method operates at the bounding-box level. Object detection is efficient and deployment-friendly, but it is inherently difficult to characterize true crack boundaries and continuity, and it must balance small-crack recall against false-positive suppression. As a result, CST-YOLO is well suited for rapid crack localization, but it does not directly provide the geometric information required in maintenance practice, such as crack width, length, and connectivity. A practical future direction is therefore to integrate CST-YOLO with downstream fine-analysis modules, where detected boxes are used as candidate regions for lightweight segmentation and local refinement, followed by skeleton extraction, connected-component analysis, and calibration-based measurement. In this way, CST-YOLO can serve not only as a real-time detector, but also as an efficient front-end for a detection–segmentation–measurement framework. Meanwhile, uncertainty estimates could further support adaptation and refinement by identifying unreliable predictions for pseudo-label correction, secondary segmentation, or manual review.

Additionally, while Poisson image blending provides an effective strategy for generating synthetic training data, a quantitative assessment of the quality of these synthesized images is currently missing from the present study. The effectiveness of the Poisson blending technique relies heavily on the visual realism of the generated images, as discrepancies between the synthetic and real data distributions may lead to model performance degradation in real-world settings. Future work will include a more rigorous evaluation of image quality using objective metrics such as Structural Similarity Index (SSIM) and Frechet Inception Distance (FID) score, in addition to subjective visual inspection. These metrics will allow for a more thorough understanding of how well the synthetic cracks resemble actual field data and whether this influences the model’s robustness across different environments. Furthermore, we plan to explore more advanced data augmentation techniques and domain adaptation strategies that better align the generated synthetic data with real-world crack distributions, thus improving cross-domain generalization. This could include integrating image synthesis with uncertainty modeling to prioritize and refine those synthetic samples that are most beneficial for model training.

In summary, future work will focus on extending CST-YOLO from an accurate real-time detector to a more reliability-oriented and engineering-interpretable framework. The key directions include uncertainty-aware learning, unsupervised domain adaptation, improved image synthesis with quantitative quality assessment, and downstream fine quantification. Through these extensions, the proposed method is expected to provide not only detection results, but also calibrated confidence information and interfaces for subsequent measurement and maintenance decision support, thereby improving its applicability in real-world bridge inspection scenarios.

## Figures and Tables

**Table 1:** Advantages and Limitations of Typical Technical Approaches for Bridge Concrete Crack Recognition.

Technical Approach	Representative Idea	Advantages	Limitations
Classification/Multi-Defect Recognition	Multi-Defect Fusion Recognition [4], Limited Data Augmentation [12]	More comprehensive semantic coverage, useful for coarse screening and alerting	Weak support for localization and quantification; class imbalance and coexisting defects can cause confusion
Object Detection	YOLO Series and Improvements [6,13,14,15], Comparison of Two-Stage Detection [16]	Fast inference, deployment-friendly, suitable for rapid localization	Difficult to delineate true crack boundaries and continuity; trade-off between small crack recall and false detection suppression
Pixel-Level Segmentation/Measurement	Pixel-Level Bridge Crack Recognition [17], Detection + Segmentation Joint [18], Segmentation Reviews [19]	Can output boundaries, beneficial for quantification of width/length, etc.	High annotation cost; prone to breaking or merging under low light/complex backgrounds; high computational resource requirements
3D Mapping and Visualization	UAV Crack 3D Visualization [9,10]	Supports full bridge localization and spatial distribution representation	Sensitive to pose/reconstruction quality; complex engineering processes, error propagation difficult to control

**Table 2:** Hardware and Software Configuration.

Experimental Environment	Configuration
CPU	Intel Xeon
GPU	NVIDIA RTX 4090
GPU memory	24 GB
Operating system	Linux Ubuntu 20.04 LTS
CUDA version	CUDA 11.8
Deep-learning framework	PyTorch 2.0.1

**Table 3:** Quantitative Results of CST-YOLO and All Baselines on the Test Set.

Detection Method	Precision	Recall	mAP@0.5	mAP@0.5:0.95	FPS	Parameter	FLOPs
Faster R-CNN	0.943	1.000	1.000	0.819	40	28.12 M	69.84 G
SSD-Lite	0.986	0.967	0.978	0.662	98	3.03 M	2.80 G
YOLOF	0.955	0.941	0.944	0.547	47	42.06 M	39.35 G
YOLOX	0.843	0.949	0.931	0.402	88	0.90 M	1.24 G
YOLOv12	0.998	0.992	0.997	0.863	185	2.57 M	6.5 G
RTDetr-ResNet50	0.133	0.589	0.201	0.084	40	42.76 M	130.50 G
CST-YOLO	0.979	0.991	0.992	0.926	139	2.74 M	8.90 G

**Table 4:** The comparison of evaluation metrics across modules.

Detection Method	Precision	Recall	mAP@0.5	mAP@0.5:0.95	FPS
YOLOv10	0.959	0.936	0.968	0.894	137
YOLOv10_LAPM	0.974	0.980	0.977	0.906	143
YOLOv10_SCS-Former	0.974	0.970	0.976	0.909	140
YOLOv10_SOFB	0.970	0.965	0.982	0.909	142
YOLOv10_LAPM_SCS-Former	0.976	0.989	0.985	0.913	139
CST-YOLO	0.979	0.991	0.992	0.926	139

**Table 5:** The comparison of evaluation metrics across models.

Model	Precision	Recall	mAP@0.5	mAP@0.5:0.95
CST-YOLO-with-PoissonBlending	0.989	0.997	0.995	0.938
CST-YOLO-without-PoissonBlending	0.979	0.991	0.992	0.922

## Data Availability

The original contributions presented in this study are included in the article. Further inquiries can be directed to the corresponding author.
